# The role of family doctors in the management of domestic violence cases – a qualitative study in Portugal

**DOI:** 10.1186/s12913-023-09501-9

**Published:** 2023-06-02

**Authors:** Diana Nadine Moreira, Mariana Pinto da Costa

**Affiliations:** 1 Institute of Biomedical Sciences Abel Salazar (ICBAS), University of Porto, Porto, Portugal; 2 Institute of Public Health of the University of Porto, Porto, Portugal; 3 Institute of Psychiatry, Psychology & Neuroscience, King’s College London, London, UK

**Keywords:** Domestic violence, Physicians, General practitioners, Primary care, Role, Management, Qualitative, Portugal

## Abstract

**Background:**

Domestic violence leads to multiple health problems in victims and their families. Family doctors are in a particularly privileged position to detect, follow up, refer and report cases of domestic violence. However, little is known about the perception of these physicians regarding their role in managing domestic violence cases.

**Methods:**

We conducted semi-structured interviews with family doctors from all regional health administrations of continental Portugal. Interviews were audio recorded, transcribed, and analysed using thematic analysis.

**Results:**

Fifty-four family doctors participated in this study (n = 39 women, n = 15 men). The themes and subthemes that emerged from the data analysis translated doctors’ broad responsibilities when approaching victims and aggressors. These included: “Implement preventive measures”, “Empower the victim leading them to recognize the violent situation”, “Detect domestic violence cases”, “Treat health problems related to violence”, “Provide emotional support”, “Refer victims to specialized services”, “Register the episode on the victims and/or aggressor clinical records”, “Incentivize the victim to report”, “Report the case to the authorities”, “Intervene with the aggressor”, “Protect other individuals” and “Follow up the patient and the process”.

**Conclusions:**

The results of this study provide an overview of the current practical approaches being adopted by physicians and may provide a base for developing new interventions to support physicians to manage cases of domestic violence.

**Supplementary Information:**

The online version contains supplementary material available at 10.1186/s12913-023-09501-9.

## Introduction

Domestic violence (DV) is a highly prevalent public health concern, with serious consequences at the individual, family, and community level [1, 2]. It is defined as any act or threat of physical, psychological, or sexual abuse perpetrated within an intimate relationship [3]. Estimates suggest that one in three woman and one in four men become victims of DV during their lifetime [4]. In 2022 the Portuguese Commission for Citizenship and Gender Equality registered the highest numbers of reports of DV in the past three years, according to the numbers provided by the Public Security Police (PSP) and the Republican National Guard (GNR), with 30,389 cases filed to these law enforcement entities [5]. The reported numbers may have been influenced by the COVID-19 pandemic and its social and economic impact, as studies have shown a decrease in reports during periods when more restrictive measures were implemented by the Portuguese government to prevent the spread of the virus [6, 7]. DV has been linked to adverse health issues in both victims and aggressors, leading to a higher utilization of health services [4, 8, 9]. Apart from direct injuries resulting from physical violence, victims experience increased rates of psychological disorders, including depression, anxiety, post-traumatic stress disorder, substance abuse, and suicidality [4, 10]. Victims may also develop functional diseases that compromise their quality of life [3]. DV has been linked to overall lower health outcomes, gastrointestinal disorders, like irritable bowel syndrome, chronic pain syndrome and gynecological diseases [9, 11]. The physical and mental impact of multiple episodes of abuse is cumulative and may manifest in victims even when they are no longer experiencing it [12]. With respect to the aggressor, DV has been linked with increased alcohol and substance abuse, depressive symptoms, post-traumatic stress, anxiety disorders, and personality disorders [10, 13, 14].

Primary health care has been recognized as an ideal setting for the detention and response to DV cases [15, 16]. Family medicine is based on a holistic, longitudinal, relationship-based approach to health care, connecting individuals with multiple services and secondary care providers. These characteristics of family medicine put doctors in a unique position to intervene with victims and aggressors. However, family doctors’ views concerning their role in managing DV cases has been under-researched. Therefore, this study aimed to investigate the following research question: “What is the perception of family doctors in Portugal regarding their role in DV cases?”

## Methods

### Study design

The findings presented in this article are part of a broader qualitative investigation concerning family doctors’ position in managing and reporting DV cases, including the investigation of the barriers and facilitators encountered by family doctors when considering presenting a report to the authorities [17].

The reported data was collected through semi-structured interviews with family doctors in Portugal. The interviews were conducted following a topic guide developed by the authors.

### Setting and participants recruitment

Participants were recruited from the five Regional Health Administrations (RHA) of continental Portugal: North, Center, Lisbon and Tagus Valley, Alentejo and Algarve. An introductory email was sent to every Family Health Unit and Personalized Health Care Unit of each of the RHAs, inviting family doctors of each institution to participate in the study. Potential participants were provided with a link that redirected them to an online questionnaire. This link was disseminated using doctors’ professional email and was shared on several social media groups and forums related to family medicine. The questionnaire included three consecutive online pages: the first was a description of the project and the authors; the second included an online consent form to be filled by the participant before proceeding to the last and third page, a sociodemographic questionnaire.

Participants’ email address was requested at the end of the online questionnaire to facilitate the scheduling of the interview. The lead author (DNM) conducted the interviews in person, when possible, or through online platforms: Zoom, Skype, or Teams, according to participants’ preference.

The only inclusion criteria was to be a specialist in family medicine currently working in Portugal. To detect possible regional differences between participants the authors aimed to conduct 10 to 20 interviews in each Regional Health Administration, based on the results of previous research on code saturation [18–20].

### Data collection and analysis

The interviews took place between July 2020 and September 2022, and were audio recorded, transcribed, and analysed through thematic analysis using the approach proposed by Braun & Clarke [21]. This method allows the researcher to identify relevant patterns and organize the data collected during the interviews to clarify their underling meaning. Transcripts were uploaded into QSR International NVivo version 12, which was used to manage and analyse the data. The analysis explored the entire dataset of the study. The analysis was inductive and based on the content of the transcripts rather than on any existing theory or hypothesis. Themes and subthemes were reviewed by both authors several times during the analysis to guarantee internal consistency between the underlying idea expressed, and the codes that generated each theme. Coding saturation was achieved, and no new codes emerged from the data. The frequency of each of the subthemes was calculated by counting how many participants addressed each.

## Results

Eighty-four doctors responded to the online questionnaire. Of these, two changed their mind and declined to be interviewed, two scheduled the interview but did not attend, and twenty-six never replied to the email to schedule the interview. In total, 54 completed the interview (12 from the North RHA, 12 from the Center RHA, 12 from the Lisbon and Tagus Valley RHA, 6 from the Alentejo RHA, and 12 from the Algarve RHA). The median duration of the interview was 23 min (IQR 9:00 to 45:00 min).

Most participants were female (72%), ranging from 30 to 65 years. The detailed sociodemographic information of the participants is presented in Table 1.

**Table 1:** Participants socio-demographic characteristics

	Characteristic	Participants (N = 54)
Gender N (%)	Female	39 (72.2)
	Male	15 (27.8)
Age N (%)	30–34 years	15 (27.8)
	35–44 years	21 (38.9)
	45–54 years	9 (16.7)
	55–64 years	8 (14.8)
	> 65 years	1 (1.9)
Sexual orientation N (%)	Heterosexual	50 (92.6)
	Homosexual	1 (1.9)
	Bisexual	2 (3.7)
	NR	1 (1.9)
Ethnicity N (%)	White	50 (92.6)
	Black	1 (1.9)
	Mixed race	2 (3.7)
	NR	1 (1.9)
Marital status N (%)	Single	7 (13.0)
	In a relationship without cohabitation	1 (1.9)
	In a relationship with cohabitation	12 (22.2)
	Married	28 (51.9)
	Divorced	5 (9.3)
	NR	1 (1.9)
Years of professional experience N (%)	1–9 years	24 (44.4)
	10–19 years	17 (31.5)
	20–29 years	4 (7.4)
	30–39 years	9 (16.7)

NR: no response

There were five themes and twelve sub-themes relating to the role of physicians. Table 2 describes these themes and sub-themes according to the different aspects of the doctors’ intervention.

**Table 2:** Themes, subthemes and their frequency

Themes	Subthemes	Number of doctors that cited each subtheme N (%)
Create awareness regarding DV	Implement preventive measures	5 (9.26)
	Empower the victim leading them to recognize the violent situation	29 (53.70)
Manage victims in the consultation	Detect domestic violence cases	15 (27.78)
	Treat health problems related to violence	4 (7.41)
	Provide emotional support	35 (6.81)
	Referrer victims to specialized services	29 (53.70)
Deal with the legal aspects of DV	Register the episode on the victims and/or aggressor clinical records	4 (7.41)
	Incentivize the victim to report	25 (46.30)
	Report the case to the authorities	21 (38.89)
Intervene with other individuals	Intervene with the aggressor	11 (20.37)
	Protect other individuals	4 (7.41)
Follow up	Follow up the patient and the process	12 (22.22)

### Create awareness regarding DV

#### Implement preventive measures

Family doctors described health promotion and disease prevention as essential aspects of their intervention. Some of the doctors interviewed affirmed to work with their younger patients implementing activities to prevent DV. The multiple consultations planned to monitor the development of children and adolescents, which in Portugal are primarily the responsibility of family doctors, are used by the doctors to address questions related to violence. Doctors seek to imbue their younger patients with the capacity to recognize aggressive behaviours as intolerable, diminishing the probability of them being part of a violent relationship, either as a victim or the aggressor.

*“The intervention shouldn’t be, I think […] shouldn’t be reactive. It should be preventive. When I have a consultation with a teenager. Normally, I don’t do it as much with children, but with teenagers, 12–13 years… my first intervention.“* (Participant 01).

Beyond this intervention, doctors also stated to use family planning and pregnancy follow-up consultations to work with couples, preparing them for the challenges of parenthood while keeping a healthy relationship.

#### Empower the victim leading them to recognize the violent situation

Most of our participants considered that frequently victims devalue their situation, perceiving episodes of violence as normal and not as a crime punishable by law. In such cases, they felt it was the doctor’s responsibility to empower the victim, providing them with key information and leading them to recognize the violence in their relationship.

*“[…] because as our society still looks at doctors in a very paternalistic way, what we say is taken into account by the patients as the truth, so if we can make this warning, right from the start, people will be more capable to recognize that they are in a violent situation, that maybe they would not otherwise have and would think it was normal.“* (Participant 33).

### Manage victims in the consultation

#### Detect domestic violence cases

Several doctors described their role as essential in the detection of DV cases. Their position as the physician following not only the victim but also other relatives, including the aggressor, the longitudinal nature of family medicine, and the degree of trust maintained with their patients puts them in a privileged position to detect DV cases.

*“I think [medical intervention] can have a great impact, on one hand because of what I’m telling you, that you know the context, you know the people close to the possible victim. We also have the consultation time, which doesn’t have to be long, a short time, because you can see in an interview how the patient behaves with the person that he lives with, right?“* (Participant 11).

Doctors put emphasis not only on their clinical knowledge, allowing them to establish a connection between signs and symptoms presented by the victim and possible episodes of violence, but also their judgment regarding family dynamics which may be a crucial factor for detection. The importance of domiciliary consultations as a means to observe their patients more closely in their family context was also described.

#### Treat health problems related to violence

One of the most evident roles of family doctors is treating possible physical or psychological injuries resulting from the abuse. At this level, doctors also described the importance of recognizing the link between specific pathologies with DV, treating them, and the underlying cause.

*“If the family doctor understands or makes this connection between these symptoms, these pathologies with violence, explaining to… to the victim this reasoning … in a way they understand… eh, because we can’t just treat here, right? Has… has a gastritis let me just give a proton-pump inhibitor because that alone won’t be enough, right?“* (Participant 04).

#### Provide emotional support

Doctors frequently emphasized the psychological consequences of the abuse. By establishing a trustworthy relationship with their patients, many doctors believed that they may function as an important, or sometimes the only, source of support for the victim. In such cases, the doctors need to manage their role as health professionals while providing emotional support and being open to helping the victim psychologically.

*“So, we have to almost move away from our position as doctors and be there… really like a friend with whom someone is venting.“* (Participant 08).

#### Refer victims to specialized services

Family doctors often have to communicate with other services and medical specialities to answer patients’ needs. In the context of DV, doctors frequently referred victims to psychology and psychiatric consultations, social services, support institutions, and in the case of minors, to the Commission for the Protection of Children and Young People (CPCJ). In most cases, the interviewed physicians did not see these referrals as a way to excuse themselves of providing further care by the family doctor but as a way to get an additional source of support to the victim.

*“And create a structure, and we have other easiness of signalization [to] other structures, social services, psychologists […] help the person at that moment for some support structures.“* (Participant 23).

### Deal with the legal aspects of DV

#### Register the episode on the victims and/or aggressor clinical records

Some doctors expressed concern about keeping detailed clinical records. This concern stems from the potential need to use these records as evidence in the judicial process against the aggressor.

*“What I always try to do is, since they are going to search, eh legally for any information, I try to write as much as possible in the clinical record, this is the most, I think this is the most important.“* (Participant 32).

#### Incentivize the victim to report

Regarding the report of DV cases to the authorities, doctors frequently preferred as an initial approach to incentivize the victim to be the one making the report. Doctors considered beneficial to work with the victim, giving them the tools to leave the abusive relationship, creating a safe exit plan, and preparing them for possible consequences of the report. Deciding if and when to present a report would allow the victim to regain control over the situation, giving them a sense of security.

*“I think that the path is to start by encouraging the victim to be the one to report.“* (Participant 05).

Doctors also considered the possibility of involving other family members of the victim in the reporting since they may provide care to multiple elements of the same family.

#### Report the case to the authorities

When the victim refuses to report the violence to the authorities, many doctors mentioned that they reported themselves. This decision was often influenced by their assessment of the risk incurred by the victim while maintaining a violent relationship. However, some doctors assumed a more proactive stance from the moment of detection to comply with mandatory reporting legislation.

*“[…] I usually say: “Look, this is a public crime. What you are telling me, I cannot, here I’m not subject to professional secrecy, I mean, I have to report it, that’s it. […] Therefore, we can collaborate the two of us […] and report it together. I don’t mind being with you. But I have to report it.“* (Participant 15).

When the doctor filled a report, most of our participants stated that they would still inform the victim previously to the report so they could be alert and prepared for possible consequences, namely retaliation by the aggressor. However, some preferred doing an anonymous report without the victims’ knowledge to preserve their doctor-patient relationship with the victim and the aggressor.

### Intervene with other individuals

#### Intervene with the aggressor

As family doctors, participants frequently stated their duty of care for both the victim and the aggressor. This position was perceived as advantageous in some cases since it could facilitate detection but also posed some difficulties, especially in cases where doctors were confronted with different versions of the same alleged episode of violence, not knowing in whom to believe and compromising their intervention. In some cases, doctors considered that an intervention with the aggressor and family therapy could be beneficial and part of their role as health professionals.

*“Helping the patient and eventually helping the aggressor, because in family medicine we can also send them to family therapy, or sometimes promote family conciliation.“* (Participant 39).

In this context, doctors highlighted cases where the aggressor had a mental disorder, not being completely aware of the severity of their actions. In such cases, the intervention by the family doctor would have a curative intention, eventually leading to the cessation of the abuse.

*“We are doctors of both, aren’t we? I had a man who had never been aggressive before, but lately was having some actions that the wife described as violent, especially verbal. Our suspicion is frontotemporal dementia. In this case, we have to treat the man.“* (Participant 24).

#### Protect other individuals

Apart from the victim and the aggressor, family doctors frequently care for other elements of the family. Taking this into account, some doctors referenced the need to sometimes extend their intervention and protect other individuals involved in the violence dynamic. In this regard, doctors described paying particular attention to children even when they were not the direct victims of the abuse but would be present, possibly assimilating violent behaviours as normal.

*“It is important to understand who is suffering with the situation. It’s not only the victim, the children and other family members may also be suffering, and we have to provide help to everyone.“* (Participant 25).

### Follow up

#### Follow up the patient and the process

Doctors highlighted the continuity of care characteristic of family medicine that allows them to follow the DV situation until its resolution. Doctors reaffirmed their availability to intervene with the victim and the aggressor at different moments according to the necessities voiced by both.

*“That’s it, follow up. Afterwards, I always ask, “how is the situation? Have you already talked with the psychologist? Have you already talked with the public prosecutor? How is the situation?“ […] It is called an attentive spectator, you know? I keep scheduling normal routine consultations and keep asking. “Has anything happened yet? Are you waiting for any development?“* (Participant 16).

## Discussion

### Key findings

Family doctors considered their intervention essential in managing DV cases, assuming different and multiple roles towards victims and aggressors. This included the prevention of DV by working with their patients, especially young people, to address social and cultural beliefs that lead to the normalization of violent behaviour. According to doctors, this acceptance of violence may also lead victims to undervalue their situation, compromising their readiness to ask for help. In these cases, doctors frequently highlighted the importance of providing critical information to their patients, leading them to acknowledge their victim status and the need for a resolution.

Family doctors recognized that the continuous and close relationships established with their patients put them in a privileged position to detect, refer, and follow up DV cases. Detection is essential in allowing a holistic approach to patient’s complaints and the support of victims and their families. Doctors also underline their role as healthcare providers for both victims and aggressors. This position could facilitate the detection of DV but is sometimes challenging to manage, especially when doctors are confronted with different accounts of the same alleged violent episode. Some doctors emphasize the importance of intervening with families and the aggressor, especially in cases where the violent behaviour was possibly routed to a psychiatric or neurological disorder.

Doctors assume two different positions regarding the reporting of DV to the authorities. Initially, most doctors try to incentivize the victim to make the report by providing essential information and working with the victim to establish a safe plan to escape the abusive relationship. However, if the victim does not take action, many doctors assume the decision to report the crime, especially in situations that put the life and health of the victim in danger.

### Comparison with the literature

Our study describes the multiple roles assumed by family doctors while managing a DV case. Doctors see themselves as key elements for addressing and combating social problems like DV. This willingness to take action is not universally present in similar studies. In a survey conducted in the USA, 13.3% of health professionals affirmed that it was not their responsibility to deal with problems related to DV, showing low confidence in the approach and management of patients suffering abuse [22].

Our participants recognized the importance of being able to detect cases of DV to allow for early intervention. This is especially relevant since victims rarely disclose the abuse to health professionals, even when resorting to consultations for the treatment of depression or anxiety issues related to DV [23]. Victims are more willing to disclose when the health professional asks directly in a non-judgemental way [8, 24]. However, doctors often find several barriers when questioning patients about DV, leading to low screening rates [25, 26]. Doctors frequently mention lack of training, guidelines, and well-defined or established referral pathways, believing they do not have the skill or sufficient time to provide an adequate response. Some doctors also fear possible retaliation from the aggressor against the victim or themselves and believe that the victim will not want to disclose the abuse choosing to stay in the abusive relationship [16, 27, 28]. These barriers are also experienced by other professionals dealing with DV cases including social workers, law enforcement professionals, other health professionals and medical specialists [29–32].

Having identified a case of DV, the family doctors in our study cited multiple ways to respond to victims. Besides treating physical and psychological symptoms related to the abuse, doctors see themselves as a source of emotional support to the victim beyond their professional relationship. Previous studies demonstrate that abused women did not consider their family doctor particularly helpful in resolving their problems but valued their empathic counseling style, the validation of their situation, and the option for additional referral to more specialized services [23, 33]. This empathic posture is reinforced by the Word Health Organization and the Portuguese General Directorate for Health (DGS) recommendations to health professionals responding to DV: “listen; inquire about needs; validate patients experiences; assess safety and risk; record; refer; and offer ongoing support” [34, 35].

Our participants also highlighted the need to write detailed clinical records documenting the history presented by the alleged victim and their observations of any physical or psychological signs of abuse. The importance of these records is substantiated by their potential use as proof in a future court case against the aggressor. A Spanish retrospective study analyzing 197 clinical records related to DV revealed multiple errors: unreadable or missing information, namely victims’ personal data and doctors’ identification. In 7.1% of the cases, victims’ identification was not possible based on clinical records [36]. This unsatisfactory quality of clinical records compromises any potential future juridical process.

Our participants often cited the need to intervene with aggressors. Portuguese guidelines regarding DV cite aggression and impulse control management programs and self-care groups as acceptable options [35]. However, there are very few studies to substantiate the efficiency of any intervention with aggressors, although psychological therapies directed to violence and alcohol abuse showed some promise [37].

Finally, most of the doctors interviewed in our study reinforced their roles as someone that can incentivize and support the victim to report the crime to the authorities. When the victim would not report the abuse, many of the doctors interviewed assumed this responsibility for reporting the crime. This dichotomic approach to reporting was also observed in studies in other countries such as Turkey, the USA and France [38–41]. A cross-sectional survey with primary care professionals in Turkey showed that 64.3% of doctors report DV cases encountered in their clinical practice. When opting not to report, doctors would incentivize the victim to do so [38]. Other survey conducted in California showed that 59% of the physicians inquired may choose not to present a report to the authorities even when working under mandatory report laws. However, more than 90% of these doctors agree that a report to the police must be done regardless of the law in specific circumstances, such as the involvement of children, pregnancy, repeated complaints, or an immediate threat to the victims’ safety [40]. In France, specific guidelines were developed which clarify in what situations breaking professional secrecy is justified [41].

### Implication of the findings for future practice and research

This study shows that family doctors in Portugal are willing and capable of assuming multiple roles in DV cases. In 2014, the Portuguese General Directorate for Health published a document addressing the approach, diagnosis, and intervention of interpersonal violence by health services [35]. The overall roles and responsibilities described by the doctors in our study mimic the steps underlined in this document as the fundamental approach to DV cases. However, theoretical knowledge does not equal a practical application, and many studies have revealed multiple barriers encountered by healthcare professionals when dealing with victims and aggressors [23, 37, 42, 43]. The willingness of our participants to tackle DV in their clinical practice should prompt governmental institutions and medical associations to support healthcare professionals by providing them with the fundamental knowledge and training required to address DV. More studies are needed to thoroughly understand the factors contributing to DV, and the tactics healthcare workers adopt to respond to victims, aggressors, and their families. The efficiency of different approaches in protecting the victim and leading to the just punishment and hopeful rehabilitation of the aggressors needs to be further addressed. Guidelines that serve as the base for a comprehensive approach to DV should be disseminated, limiting each physician’s adoption of individual subjective practices.

### Strengths and limitations

This is the first study in Portugal exploring family doctors’ views regarding their role and responsibilities in DV cases. By investigating these professionals’ opinions, we went beyond theoretical guidelines and acquired a more practical understanding of family doctors’ practices when faced with DV cases.

The inclusion of doctors from each of the five RHA’s of continental Portugal strengthened our study by providing a comprehensive view not limited by social, regional or cultural contexts. The option for one-to-one interviews and an inductive approach to the data allowed for the translation of various participants’ points of view.

The study presents also some limitations. Participants were not questioned regarding their personal experiences with DV, which could influence their perception of what should be their role and professional approach.

## Conclusions

This study investigated the perception of family doctors in Portugal regarding their roles and responsibilities while managing cases of DV. Family doctors see themselves as a source of support for the victims, providing relevant information that may lead them to grasp the violence in their relationship and incite change. Beyond their work with the victims, doctors recognize their responsibility to care for the aggressor and other elements of the family, working frequently with other institutions and specialized health services. Doctors often considered presenting a report to the authorities, particularly when representing a high risk to the health or life of the victim. The findings from our interviews to family physicians in Portugal provide an overview of current practices across the country and are concurrent with international practice.

## Electronic supplementary material

Below is the link to the electronic supplementary material.

Supplementary Material 1

Supplementary Material 2

Supplementary Material 3

Supplementary Material 4

## Data Availability

The datasets analysed available from the corresponding author upon reasonable request.
