# Supplementary material for: The role of family doctors in the management of domestic violence cases – a qualitative study in Portugal
Source: BMC Health Serv Res. 2023 Jun 2;23:571. doi: 10.1186/s12913-023-09501-9 (PMC10237072; doi:10.1186/s12913-023-09501-9)
Supplement: Supplementary file 1 — Supplementary Material 1 [file 12913_2023_9501_MOESM1_ESM.docx]

**Appendix 1** – Interview Guide

**Interview Guide**

The following questions serve as a guide for the interviewer during the semi-structured interview. It is optional to follow the order of questioning presented below. The interviewer may reformulate the questions to create a logical occurrence with participants’ answers.

1. **In your opinion, what defines domestic violence?**

- Different types of violence – psychological, physical, sexual;
- Individuals involved – between couples, in dating, other members of the household;
- Reference to criminality;

1. **What do you think about the mandatory reporting of domestic violence cases to authorities?**

- Implications for the general population;
- Implications for doctors;

1. **In your professional experience, have any of your patients ever told you they are a victim of domestic violence?**

(If you have not had direct experience with domestic violence cases, have you been aware of any from your colleagues?)

**3.1. What did you do in that situation?** / What was done in that situation?

**3.2. What difficulties did you encounter?** / What problems were encountered?

**3.3. If it were today, how would you proceed?** / If it was you in that position, how would you proceed?

1. **In your opinion, how can the intervention of family doctors impact the life of domestic violence victims?**

- Health status;
- Quality of life;
- Recurrence of violence episodes;
- Safety;

1. **What factors would motivate you to report a domestic violence case to the authorities?**
2. **What factors would make it difficult for you to report a domestic violence case to the authorities?**
