# Supplementary material for: The role of family doctors in the management of domestic violence cases – a qualitative study in Portugal
Source: BMC Health Serv Res. 2023 Jun 2;23:571. doi: 10.1186/s12913-023-09501-9 (PMC10237072; doi:10.1186/s12913-023-09501-9)
Supplement: Supplementary file 2 — Supplementary Material 2 [file 12913_2023_9501_MOESM2_ESM.docx]

**Appendix 2 –** Sociodemographic questionnaire

**Sociodemographic questionnaire**

We invite you to answer these questions to better understand the diversity of the participants in our study. All the information is confidential. Please write your answer in the correct space or select one or more of the available options that better describe you.

**Age**: ________ years

**Sex**: □ Female; □ Male; □ Other. Which? _____________________________________

**How would you describe your sexual orientation**? □ Heterosexual; □ Homosexual;

□ Bisexual; □ Other. Which? _______________________________________________

**How would you describe your ethnicity**? □ White; □ Black; □ Asian; □ Romani; □ Mixed race; □ Other. Which? ___________________________________________________

**How would you describe your marital status?** □ Single; □ In a relationship without cohabitation; □ In a relationship with cohabitation; □ Married; □ Divorced;□ Widow(er); □ Other. Which? _______________________________________________

**For how many years have you been working as a family doctor**?_______years

**Currently you are working in a**: □ City; □ Town; □Village; □ Hamlet;

**In which RHA do you currently work?** _____________________________________

Please provide us with your **email address** to facilitate further contact and the scheduling of the interview: _______________________________________________
