# Supplementary material for: The role of family doctors in the management of domestic violence cases – a qualitative study in Portugal
Source: BMC Health Serv Res. 2023 Jun 2;23:571. doi: 10.1186/s12913-023-09501-9 (PMC10237072; doi:10.1186/s12913-023-09501-9)
Supplement: Supplementary file 3 — Supplementary Material 3 [file 12913_2023_9501_MOESM3_ESM.docx]

**Appendix 3** – Themes, sub-themes and illustrative quotes related to the “Role of the physician”

| **Create awareness regarding DV** | |
| --- | --- |
| Implement preventive measures | *"The intervention shouldn't be, I think […] shouldn't be reactive.* ***It should be preventive****. When I have a consultation with a teenager. Normally, I don't do it as much with children, but with teenagers, 12-13 years… my first intervention."* (Participant 01) |
|  | *“As in* ***everything, in prevention****. No doubt. In prevention, addressing family planning in consultations. They are a good space to talk again in these situations.”* (Participant 37) |
| Empower the victim leading them to recognize the violent situation | *“[…] from the start* ***patients trust us****… and trust our opinion and if there are people that think that domestic violence is like that because it is like that, because it has always been like that […]. If for example a doctor, or a nurse, or… saying that* ***it is not normal and that there are solutions****, maybe that person will be more willing to change, right?* (Participant 05) |
|  | *"[…] because as our society still looks at doctors in a very paternalistic way,* ***what we say is taken into account by the patients as the truth****, so if we can make this warning, right from the start, people will be more capable to recognize that they are in a violent situation, that maybe they would not otherwise have and would think it was normal."* (Participant 33) |
| **Manage victims in the consultation** | |
| Detect domestic violence cases | *"I think [medical intervention] can have a great impact, on one hand because of what I'm telling you, that you know the context,* ***you know the people close to the possible victim****. We also have the consultation time, which doesn't have to be long, a short time, because* ***you can see in an interview how the patient behaves*** *with the person that he lives with, right?"* (Participant 11) |
|  | *“Those couples in which they accompany a lot the woman to the consultation and are very sweet and such, usually I suspect. Because there is usually jealousy behind it, isn’t there? Then I try to deconstruct a little bit, or separate them […]. I catch her [the victim] alone inside and try, and she already has* ***the confidence to be able to talk****.”* (Participant 15) |
| Treat health problems related to violence | *"If the family doctor understands or makes this* ***connection between these symptoms, these pathologies with violence****, explaining to… to the victim this reasoning … in a way they understand… eh, because we can't just treat here, right? Has… has a gastritis let me just give a proton-pump inhibitor because that alone won't be enough, right?"* (Participant 04) |
|  | *“At the level of psychological support, isn’t it? At the level of our* ***intervention as a conversation and through pharmacological care*** *too. Yes if, when necessary.* (Participant 20) |
| Provide emotional support | *“The family doctor has a role that can be very important, and if you create* ***a trusting relationship with the victim****, I think that can be a huge support”* (Participant 04) |
|  | *"So, we have to almost move away from our position as doctors and be there… really like* ***a friend with whom someone is venting****."* (Participant 08) |
| Referrer victims to specialized services | *"And create a structure, and we have other* ***easiness of signalization [to] other structures, social services, psychologists*** *[…] help the person at that moment for some support structures." (Participant 23)* |
|  | *“And that in a way if we are also able to* ***articulate with the network*** *[of possible referrals]* ***that we have around us,*** *we can be an asset to the victim.”* (Participant 47) |
| **Deal with the legal aspects of DV** | |
| Register the episode on the victims and/or aggressor clinical records | *“That’s it, we can do an evaluation and* ***good clinical records****, in case there are questions, we write everything well, so that if the victim wants to report to the authorities, there are traces or signs of what happened.”* (Participant 14) |
|  | *"What I always try to do is, since they are going to search, eh legally for any information, I try to* ***write as much as possible in the clinical record****, this is the most, I think this is the most important."* (Participant 32) |
| Incentivize the victim to report | *"I think that the path is to start by* ***encouraging the victim to be the one to report****." (Participant 05)* |
|  | *“On the other hand, I think we are able to have other ways of involving people from the household who could even help the victim with the report without being us doing it.”* (Participant 23) |
| Report the case to the authorities | *“[…] I usually say: "Look, this is a* ***public crime****. What you are telling me, I cannot, here I'm not subject to professional secrecy, I mean, I have to report it, that’s it. […] Therefore, we can collaborate the two of us and report it together. I don't mind being with you. But* ***I have to report it****." (Participant 15)* |
|  | *“And if the person does not intend to file a complaint, for whatever [reason], yes I think* ***we should do it ourselves****.”* (Participant 33) |
| **Intervene with other individuals** | |
| Intervene with the aggressor | ***"We are doctors of both, aren’t we?*** *I had a man who had never been aggressive before, but lately was having some actions that the wife described as violent, especially verbal. Our suspicion is frontotemporal dementia. In this case, we have to treat the man."* (Participant 24) |
|  | *"****Helping the patient and eventually helping the aggressor****, because in family medicine we can also send them to family therapy, or sometimes promote family conciliation."* (Participant 39) |
| Protect other individuals | *"It is important to understand* ***who is suffering with the situation****. It's not only the victim, the children and other family members may also be suffering, and we have to provide help to everyone."* (Participant 25) |
|  | *“I think that what is essential from our part is perhaps to understand what drains the psychological wellbeing that the person maybe incurring, the way in which they are dealing with the situation and the* ***repercussions that this may have in other family members****, for example children or grandparents who live in the same house and that may be suffering from this. It’s more about* ***paying more attention to others****.”* (Participant 42) |
| **Follow up** |  |
| Follow up the patient and monitor the process | *“But since there was no active violence in that moment, there was no interference on my part, I just* ***let open the possibility of talking to me if necessary****.”* (Participant 09) |
|  | *"That's it,* ***follow up****. Afterwards, I always ask, "how is the situation? Have you already talked with the psychologist? Have you already talked with the public prosecutor? How is the situation?" […] It is called* ***an attentive spectator****, you know? I keep scheduling normal routine consultations and keep asking. "Has anything happened yet? Are you waiting for any development?" (Participant 16)* |
|  |  |
