# Supplementary material for: The role of family doctors in the management of domestic violence cases – a qualitative study in Portugal
Source: BMC Health Serv Res. 2023 Jun 2;23:571. doi: 10.1186/s12913-023-09501-9 (PMC10237072; doi:10.1186/s12913-023-09501-9)
Supplement: Supplementary file 4 — Supplementary Material 4 [file 12913_2023_9501_MOESM4_ESM.docx]

**Appendix 4** – Consent Form

**Consent Form**

I received a detailed explanation regarding the project, its theoretical base, aims, and methodology, which I understand and accept. I had the opportunity to ask questions about the project that were answered, and I understood those answers.

My participation is entirely voluntary. I know that I can choose to leave the project and have the previously provided information removed at any moment, up to one month after the interview, without having to give any explanation. I also know that I may choose not to answer any particular question.

I understand the information provided is anonymous and confidential, and I will be given a participant number. I understand that the data I provide may be used in future presentations and publications.

Therefore,

□ I accept to participate in the research project.

□ I accept that my interview is audio-recorded.

□ I accept that my interview is transcribed by the researcher.

□ I accept the use of extracts from my interview as anonymized citations in future presentations and publications.

□ I accept that my sociodemographic information can be compiled with those of the other participants and that eventual conclusions can be reported in future presentations and publications.

□ I accept that the researcher can keep the materials collected (interview transcriptions and the sociodemographic questionnaire answers) for future analyses and publications.
